# Mechanical osteoarthritis of the hip in a one medicine concept: a narrative review

**DOI:** 10.1186/s12917-023-03777-z

**Published:** 2023-10-24

**Authors:** I. Tomé, S. Alves-Pimenta, R. Sargo, J. Pereira, B. Colaço, H. Brancal, L. Costa, M. Ginja

**Affiliations:** 1 Department of Veterinary Sciences, University of Trás-Os-Montes E Alto Douro, Vila Real, 5000-801 Portugal; 2 CECAV, Centre for Animal Sciences and Veterinary Studies, Associate Laboratory for Animal and Veterinary Science - AL4AnimalS, University of Trás-Os-Montes E Alto Douro, Vila Real, Portugal; 3 Department of Animal Science, University of Trás-Os-Montes E Alto Douro, Vila Real, Portugal; 4 Clínica Veterinária da Covilhã, Covilhã, 6200-289 Portugal

**Keywords:** Hip mechanical osteoarthritis, Hip dysplasia, Human, Dog, Animal models

## Abstract

Human and veterinary medicine have historically presented many medical areas of potential synergy and convergence. Mechanical osteoarthritis (MOA) is characterized by a gradual complex imbalance between cartilage production, loss, and derangement. Any joint instability that results in an abnormal overload of the joint surface can trigger MOA. As MOA has a prevailing mechanical aetiology, treatment effectiveness can only be accomplished if altered joint mechanics and mechanosensitive pathways are normalized and restored. Otherwise, the inflammatory cascade of osteoarthritis will be initiated, and the changes may become irreversible. The management of the disease using non-steroidal anti-inflammatory drugs, analgesics, physical therapy, diet changes, or nutraceuticals is conservative and less effective. MOA is a determinant factor for the development of hip dysplasia in both humans and dogs. Hip dysplasia is a hereditary disease with a high incidence and, therefore, of great clinical importance due to the associated discomfort and significant functional limitations. Furthermore, on account of analogous human and canine hip dysplasia disease and under the One Medicine concept, unifying veterinary and human research could improve the well-being and health of both species, increasing the acknowledgement of shared diseases. Great success has been accomplished in humans regarding preventive conservative management of hip dysplasia and following One Medicine concept, similar measures would benefit dogs. Moreover, animal models have long been used to better understand the different diseases’ mechanisms. Current research in animal models was addressed and the role of rabbit models in pathophysiologic studies and of the dog as a spontaneous animal model were highlighted, denoting the inexistence of rabbit functional models to investigate therapeutic approaches in hip MOA.

## Background

Human and veterinary medicine have historically shown areas of convergence and overlap between fields like neurology, oncology, musculoskeletal or infectious diseases [1]. Mechanical Osteoarthritis (MOA), as a musculoskeletal condition, is characterized by a gradual complex imbalance between cartilage loss, derangement, and production [2–4], representing one of the main joint pathologies in mammals [5]. MOA is a determinant factor for the development of hip dysplasia (HD) in both humans and dogs [6], being also described in domestic cats [7, 8]. HD is a hereditary disease of great clinical importance in the human and canine species [9, 10], due to the associated discomfort and functional limitations [6]. HD in the feline species clinical signs does not seem to reach socially alarming proportions [7, 8]. The pathogenesis of dog OA closely resembles the primary disease in humans [11], despite many questions remaining unanswered regarding this poorly understood entity [9, 10]. Some genome-wide association studies in dogs identified single nucleotide polymorphisms that were linked with canine HD [12, 13] and the intronic deletion in the fibrillin-2 gene in the fibrous joint capsule was associated with canine hip laxity [13]. Similar studies were performed in humans and several genes were found to be related to HD development, such as CX3CR1 [14], GDF5 [15] and CTBP2 [16]. Moreover, joint anatomical and disease physiopathology similarities on both species associated with the spontaneous and natural occurring disease in dogs [17], greater prevalence of HD, faster progression to OA, rapid generational turnover, large litter size [18], and lower research costs comparatively to humans, may allow a foreseeable comparison of the natural history of HD in a shorter period of time [17]. Therefore, merging human and veterinary research fields, on account of analogous human and dog diseases and under the One Medicine concept, could improve the well-being and health of both species, increasing the acknowledgement of shared diseases [11, 19]. Additionally, small and large animal models have been employed in MOA research for decades [20].

The main aim of this paper is to provide an overview of the scientific developments that have taken place in the veterinary and human medical fields, regarding hip MOA and dysplasia, emphasizing their similarities, health challenges and shared risks, with the main purpose of establishing a bridge and create synergies with mutual benefits for both species.

This is a narrative review and a comprehensive, critical, and objective analysis of the current knowledge of the human and dog hip MOA, exploring the perspective of the One Medicine concept. The review starts with scientific insight into MOA physiopathology. In the subsequent sections, human congenital and canine HD similarities and differences will be discussed, as well as the therapeutic approach for disease prevention and treatment in both species. Ultimately, the importance of animal models in the study of MOA and their role in the enhancement of current knowledge is presented, emphasizing the value of the dog as a natural animal model of human MOA in a One Medicine concept.

## Main text

### Mechanical osteoarthritis

MOA is generally known as degenerative joint disease or osteoarthrosis [2–4]. It has its onset in cartilage mechanical overload, which is responsible not only for cartilage wear and tear but also for signalling mechanosensitive pathways that drive proteases to initiate the mechanism of joint breakdown [21, 22]. Hip MOA represents one of the main articular pathologies in mammals [5], having special clinical importance in humans and dogs due to the associated discomfort and significant functional limitations [6]. The disease has a worldwide impact and in humans is estimated that 240 million people are affected by this limiting condition [23], reaching an overall prevalence of 10,9% in both genders [24]. In the dog, it is projected that 20% of the dog population over 1-year-old is affected by hip MOA [25] and in some breeds, it can be present in more than 60% [26]. The social importance of the disease has increased over the years due to a combination of several risk factors, namely obesity, increased life expectancy, as well as a greater global concern with well-being and quality of life [23].

Osteoarthritis (OA) is either primary or secondary. In humans, the primary form is the most frequent condition and the secondary the less common type [27], whereas in dogs the opposite is observed [28]. The primary condition is essentially defined as idiopathic, with no identifiable underlying cause, attributed to a deficient biosynthesis and cartilage structure [28, 29]. Ageing represents an inherent factor in this type of OA, being determinant in the cartilage matrix composition and chondrocyte function [27]. The secondary type is triggered by other underlying conditions such as joint overload associated with obesity [23] or mechanical arthropathies, such as trauma, atypical stress, and anatomic malformations, which promote MOA development [28, 29].

Articular cartilage has distinctive compressive and viscoelastic properties, creating a deformable tissue capable of absorbing the load-bearing impact and decreasing the friction of articular surfaces [28, 30]. The extracellular matrix provides the mentioned properties, being the proteoglycans, type II collagen, and hyaluronan part of its composition, along with a high-water content [30, 31]. Since cartilage is an avascular tissue, the nutrition of chondrocytes and repair elements are essentially provided through the synovial blood supply [32, 33]. The synovial fluid, whose main function is to diminish the friction between articular surfaces [34], contains high levels of hyaluronan, also known as hyaluronic acid or hyaluronate [31]. Synoviocytes ensure the synthesis of synovial fluid [31, 34].

**Fig. 1:**
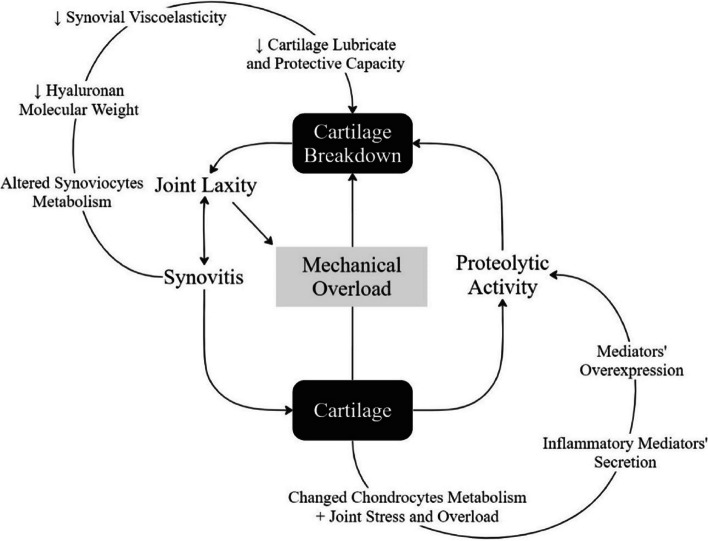
Schematic representation of the hip mechanical osteoarthritis pathogenesis [35, 36]

Products of cartilage breakdown released into the synovial fluid, due to excessive catabolism, favour the initiation and maintenance of synovitis. When synovitis is present, inflammatory mediators and infiltrating leukocytes increase vascular permeability and plasma concentration, reducing hyaluronan concentration in the synovial fluid. This hyaluronan dilution decreases the viscoelasticity of the synovial fluid and, consequently, its capacity to protect and lubricate the cartilage [35] (Fig. 1). The main inflammatory mediators, cytokines (interleukin-1 and -6, and tumour necrosis factor), matrix-degrading enzymes, nitric oxide, and reactive oxygen species, secreted by synoviocytes and chondrocytes and enhanced by joint stress and overload, play a crucial role in the onset and progression of MOA [36]. Their overexpression promotes the production of cartilage breakdown enzymes, leading to continuous decay of the synovial membrane (synovial inflammation, hyperplasia and fibrosis), articular cartilage (reduction in proteoglycan, hyaluronic acid and collagen, and cartilage loss), and subchondral bone (attenuation of mineral accretion) [28, 36, 37]. Excessive proteolytic activity is attenuated by the endogenous inhibitors present in the synovial fluid [38]. However, cytokines and reactive oxygen species are freely scattered into cartilage and dysregulate the proteoglycan and type II collagen biosynthesis [39]. These cytokines can also begin the production of catabolic proteinases and more free radicals and cytokines, contributing to additional matrix destruction [35]. MOA is then an imbalance between catabolic and anabolic processes, when extensive defects surpass cartilage repair capacity that would otherwise be regenerated by hyaline cartilage, making the damage permanent [40]. Although the mechanism that triggers cartilage degradation is still unknown, environmental, genetic, hormonal [32], biomechanical, and metabolic [30] components may play a critical role [30, 32].

In the light of present knowledge two main components, inflammatory and mechanical, are described regarding the development of MOA [41]. Still, several potential MOA phenotypes, such as clinical signs and structural damage are linked with mechanics [42]. The articular disease is believed to have a mechanical component when a pathophysiological response to a mechanical injury is present, leading to an uneven load bearing in localized areas of the joint [21]. The inflammatory response would then be considered an attempt by the joint to fix the atypical stress distribution and repair the osteoarthritic injury [21, 43], leading to an increase in cytokines, matrix-degrading enzymes, and free radicals [35]. The risk of developing MOA can be divided into cases where an abnormal force distribution is translated into an excessive mechanical stress spread through a healthy articulation, due to overweight [21, 44], joint incongruity, changes in gait patterns, and isolated/ repeated overload [44]; or in cases where the joint that has lost its mechanical-protective mechanisms [21]. Mechanical protection is provided by a steady joint [45], a strong supportive musculature, and undamaged gait reflexes [46].

MOA is a disease characterized by chronic pain, lameness [47–49], stiffness, joint effusion, and crepitus associated with pathological changes in the synovial joint [49]. Animals can also experience reluctance to physical activity [28, 50] and reduced range of motion [50]. In humans and dogs, the disease is generally managed by employing a conservative approach, using non-steroidal anti-inflammatory drugs, analgesics, physical therapy, diet changes or nutraceuticals; by a non-conservative approach, or a combination of both [47]. Currently, after the inflammatory cascade starts, MOA is an incurable condition and management guidelines are focused on addressing pain and improving symptoms and overall function [23]. It should be noted that an effective MOA treatment can only be achieved if the altered joint mechanics and mechanosensitive pathways are early normalized and restored [51]. Otherwise, the inflammatory cascade of OA will be activated, and the damage can become irreversible [52].

MOA has challenged researchers and veterinary clinicians for decades [53], affecting the well-being of a great number of canine joints [54]. Multiple joint involvements are recognized in MOA physiopathology, being the hip, stifle, shoulder, and elbow the most commonly reported [11, 54]. An increased awareness of the disease pathogenesis and an early diagnosis will assist in the implementation of preventive measures [2].

In hip MOA, the fact that humans are bipeds and dogs quadrupeds, allows the dog to compensate for eventual hip abnormalities due to the dominance of the front limbs over the hind limbs [18, 55]. The load is distributed symmetrically in a proportion of 60:40 between the front and hindlimbs [18]. In case of hip abnormalities, the load is transferred to the less affected limbs (contralateral or front limbs) [18, 55], which may have an unknown effect on the development and progression of MOA, in both affected and non-affected limbs [56].

On account of anatomy, aetiology, and pathophysiology, the dog is considered the species that presents the strongest resemblance to human OA [11]. Due to these similarities between dog and human OA, combining veterinary and human research, under the One Medicine concept, could enhance the well-being and health of both dogs and humans [11, 19].

### Congenital human hip dysplasia

In humans, congenital HD, also known as developmental HD, is normally associated with an intrauterine hip developmental abnormality that becomes noticeable immediately or a few months after birth [57]. Developmental HD is deemed as a leading precursor of hip MOA [58] and in adults results in the development of a shallow acetabulum and a flattened femoral head [59]. The acetabulum depth is determined during the skeletal ossification phase by the pressure exerted by the spherical femoral head [60].

Developmental HD, depending on the population and definition, has a prevalence that can fluctuate from 0.15 [61] to 10.5 per 1000 births [62]. It has a multifactorial origin, presenting the intrauterine breech presentation, female gender, left hip, and genetic predisposition as some of the factors involved in its aetiology [63, 64]. The combination of genetic and environmental factors is responsible for the wide geographic and ethnic variation in the incidence of congenital HD [57, 63]. Regions such as Central and South Africa, Northern Canada (Eskimos), and Hong Kong in China, where people carry newborns on their backs with the hips in flexion and abduction during postnatal growth, have a lower incidence of congenital HD [57, 65] compared to regions where people swaddle babies, maintaining the hips held in extension and adduction [64]. These cultural practices work either as an efficient preventive mechanical treatment or a promotor of HD disease. The genetic nature of developmental HD has long been known due to the epidemiological association between a higher incidence and different degrees of kinship [66, 67]. More recently, the genetic susceptibility to developmental HD has been revealed through several candidate gene studies in the canine population [14, 15]. The adequate identification of targeted genes associated with developmental HD is an essential preliminary step towards the advancement of research based on recent cell-based OA therapies [68]. Moreover, recent advances have already demonstrated the potential of viral and non-viral gene therapy in disease-modifying therapeutics for OA [68].

In terms of diagnosis, early identification of HD can be assessed by physical examination using the Barlow and Ortolani manoeuvres and ultrasonography [69, 70]. The latter is used in infants up to 4 months old due to the predominant cartilaginous nature of the hip [69, 70]. In the early ultrasonographic diagnosis, the acetabular depth and shape are assessed [69]. Following this period, femoral head ossification makes the ultrasound exploration of the acetabulum unfeasible, and the hip joint is more reliably visualized on radiographs, rendering this imaging modality as the preferred tool [69]. Upon confirmation of hip instability or luxation, bracing and closed reduction (e.g. Pavlik harness), along with a hip spica cast are the proposed approaches for infants up to 6 months and from 6 to 18 months old, respectively [69–71]. If patients within 9 to 18 months old do not achieve a concentric reduction with conservative management, hip reduction surgeries are suggested [18, 69]. Femoral procedures such as varization, femoral shortening, and derotation osteotomies [18, 72] have special importance in decreasing femoral head forces and the predisposition for avascular necrosis [72]. Pelvic surgeries like triple osteotomy [18, 69, 72], juxta-articular double osteotomy [73], Salter innominate osteotomy [74], and periacetabular surgeries such as Dega transiliac osteotomy [75], Pemberton pericapsular osteotomy [76], and Bernese periacetabular osteotomy [77]), improve in general acetabular femoral head coverage, increase the cartilage weight bearing area and reduce local overload, preventing the development of OA [78]. Salvage procedures are other non-conservative options mainly used to relieve pain in cases of irreversible cartilage degeneration and to delay hip arthroplasty fitting [72].

In general, therapeutic approaches in the human hip have significantly evolved over the last few years and great success has been achieved in arresting or delaying the onset of hip MOA. For instance, the use of shoe-lifts to compensate for leg-length discrepancies when evidence of human locomotion dysfunction is present is becoming less frequently used even among non-developed societies [79]. Nevertheless, therapeutic success and human well-being could be enhanced if, in severe cases, the need to resort to a more aggressive treatment was avoided, by eliminating gradually the progression of OA over the years and not just attenuating its clinical manifestation. In this regard, there is great prospect in current research in the identification of genes associated with the development of hip OA and in cell therapy focused on targeted genes directly associated with hip OA.

### Canine hip dysplasia

Canine Hip Dysplasia is a developmental orthopaedic disease, inherited, in which an atypical development of the coxofemoral joint leads to instability and, consequently, progresses to cartilage destruction and degenerative joint disease [80, 81]. Joint instability invariably results in subsequent MOA [82]. It is particularly present in large and giant dog breeds and can reach a prevalence of 73,4%, depending on the breed [18, 83, 84]. Despite the high rate of HD genetic predisposition, the severity of the clinical and radiographic signs is subject to change by environmental factors [6, 54]. HD, being a multifactorial disease, environmental and genetic factors play an important role in its development, specifically diet, obesity, weight [54, 85], exercise, breed, skeletal ossification process, rapid growth [85], and the increment of the femoral anteversion angle [18].

Selective breeding, using radiographic phenotypic scores or estimated breeding value, aimed to reduce the occurrence of undesirable alleles in the canine population and has been the main tool employed to diminish the clinical and phenotypic manifestation of the disease [26, 86, 87]. Technological advancements in molecular analysis of canine HD have evolved in searching for genetic markers, namely quantitative trait loci associated with the main radiographic HD phenotypes [88]. However, the complexity of HD inherence and the non-specificity of quantitative trait loci regions led to the refinement of quantitative trait loci intervals using single nucleotide polymorphisms and genome-wide association studies to join the effect of multiple canine HD single nucleotide polymorphisms [89, 90]. This is the current molecular strategy to address polygenic features of HD with strong environmental components. Nevertheless, it has not been possible to obtain a feasible molecular diagnosis for HD, despite the success of this methodology in similar genetic traits, such as meat and milk production [91, 92]. The research in this field of genetic diagnosis in canine HD remains essential, enabling the introduction of genomic selection.

**Fig. 2:**
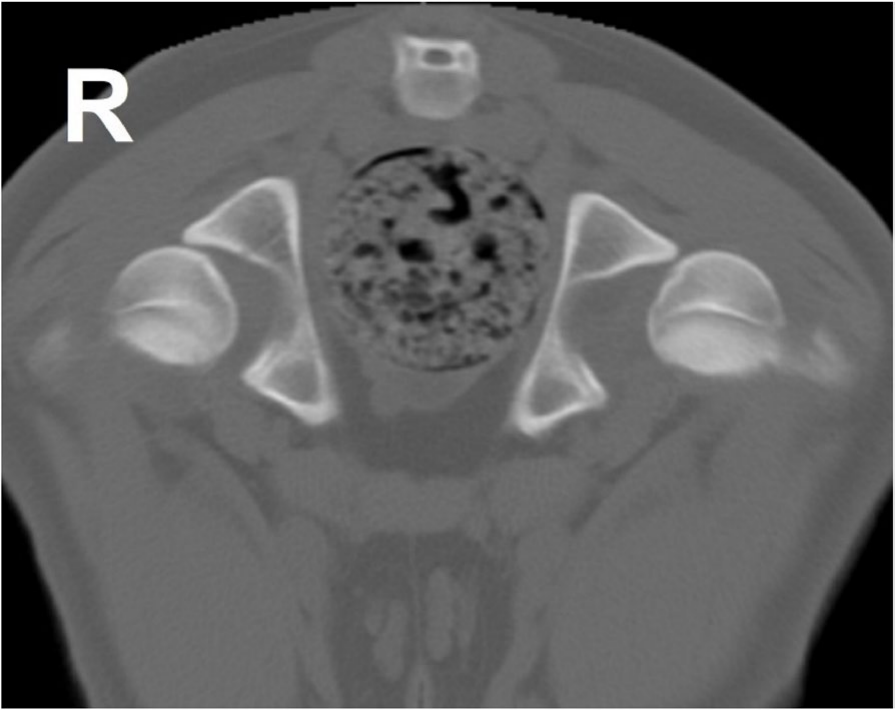
Computed tomography of the hip in a transverse plane of a 5-month-old Transmontano Mastiff dog at an early stage of hip dysplasia. An increase in hip laxity is translated into a reduced contact between the femoral head and the acetabulum under normal load bearing. The left hip joint shows a degree of subluxation more evident than the contralateral hip, resulting in a diminished femoral head-acetabulum contact area

HD is described as a deficient relationship between the acetabulum and the femoral head or joint laxity, resulting in an abnormal peak of forces and, consequently, cartilage destruction and joint inflammation [80, 93]. The motion and load bearing of the canine hip joint is subject to the structural integrity of the surrounding tissues, such as muscles, ligaments, and tendons. The distribution and magnitude of forces acting on the joint, its stability, and cartilage integrity will determine the wear and tear of the articulation [94]. Canine HD is characterized by subluxation of the femoral head [94]. The subluxation will increase the stress inflicted on the articular cartilage, decreasing the contact between the surfaces and, consequently, increasing locally the cartilage overload [94]. The resultant of forces acting on the femoral head shift from an eccentric distribution to a local distribution, being greater the wider the degree of subluxation [93, 94] (Fig. 2). Eccentric load distribution can also be responsible for shear forces when in excess, broadening the inclination angle or shifting the direction of the resultant force which contributes to the loss of articular cartilage [94].

Pain, lameness derived from a subluxation, acetabular microfractures, and capsule stretching are some of the clinical signs frequently described in immature animals with hip laxity [95]. In more severe cases, the presence of hip crepitation, fibrosis and reduced mobility are also observed [96].

**Fig. 3:**
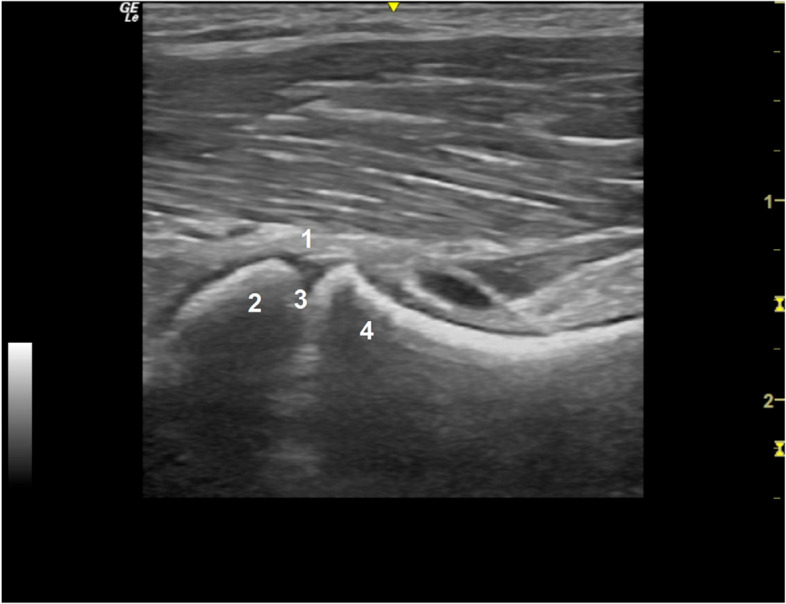
Long-axis ultrasound ventral view over the medial aspect of the femoral neck in a 2-month-old Estrela Mountain puppy without signs of joint disease: joint capsule (1), the femoral head covered by cartilage (2), the physis (3) and the metaphysis (4)

The HD recommendations are focused on early screening and selective breeding, leading to a superior hip phenotype in the next generations and gradually exerting selective pressure against the trait, aiming to reduce the disease prevalence [87]. As there is no feasible molecular modality to accurately diagnose this multifactorial disease, hip joint imaging allied with physical examination is the current methodology used in HD screening programmes, especially in young animals in the early stages of the disease [97, 98]. Early evidence of canine HD can be characterized by an increased joint laxity [96], evidenced on stress radiographs in animals from 16 to 18-weeks-old [98] and joint instability, accessed through the Ortolani manoeuvre under sedation [96, 98]. Identification of hip laxity, as an early sign of canine HD, is crucial in young animals and is assessed on stress radiographs using the distraction index [99], the laxity index [100] or the dorsolateral subluxation index [101]. The minimum age for a late diagnosis based on radiographic osteoarthritic changes is breed-dependent and is established at 12–18 months when the animals reach skeletal maturity [87]. The evaluation of radiographic OA severity should comprise a complete radiographic assessment and scoring of certain features [102]. Severe HD is characterized by hip luxation or evident subluxation, osteophytes development, a Norberg angle < 90º, remodelling of the acetabulum, a thickened femoral neck and a mushroom-shaped/ flattened femoral head [86]. The osteophyte development is correlated with OA progression and current studies have suggested a mean osteophyte growth rate of 0.0009 to 0.0036 mm per day in the dog [103, 104]. Worldwide, three main associations are considered in HD scoring: in the United States of America and Canada, the Orthopedic Foundation for Animals [105, 106]; in most European countries, South America, and Asia, the Fédération Cynologique Internationale [106, 107]; and in Britain, Ireland, New Zealand, and Australia, the British Veterinary Association/ Kennel Club [106]. Additionally, due to the non-congenital nature of canine HD and the ossification of the femoral head at 8 weeks, the ultrasonographic visualization of the acetabulum does not appear to be reliable, making radiography the preferred method for evaluating hip morphology [108]. Nonetheless, as an underdeveloped area in veterinary medicine, ultrasound studies could be used as a non-invasive tool to quantify articular volume [67, 109] or other early changes [110, 111]. The ultrasonographic anatomy of the hip joint of a juvenile dog shows a good detail of the articular and periarticular structures, displaying resemblances to the juvenile human hip, like other developmental aspects [112] (Fig. 3). Currently, paediatric hip ultrasonography is useful for screening transient synovitis, a common cause of hip pain in children from 3 to 8 years-old [113]. In the dog, the use of complementary diagnostic tools to detect pain is of utmost importance due to the inability of animals to objectively communicate slight degrees of discomfort.

**Fig. 4:**
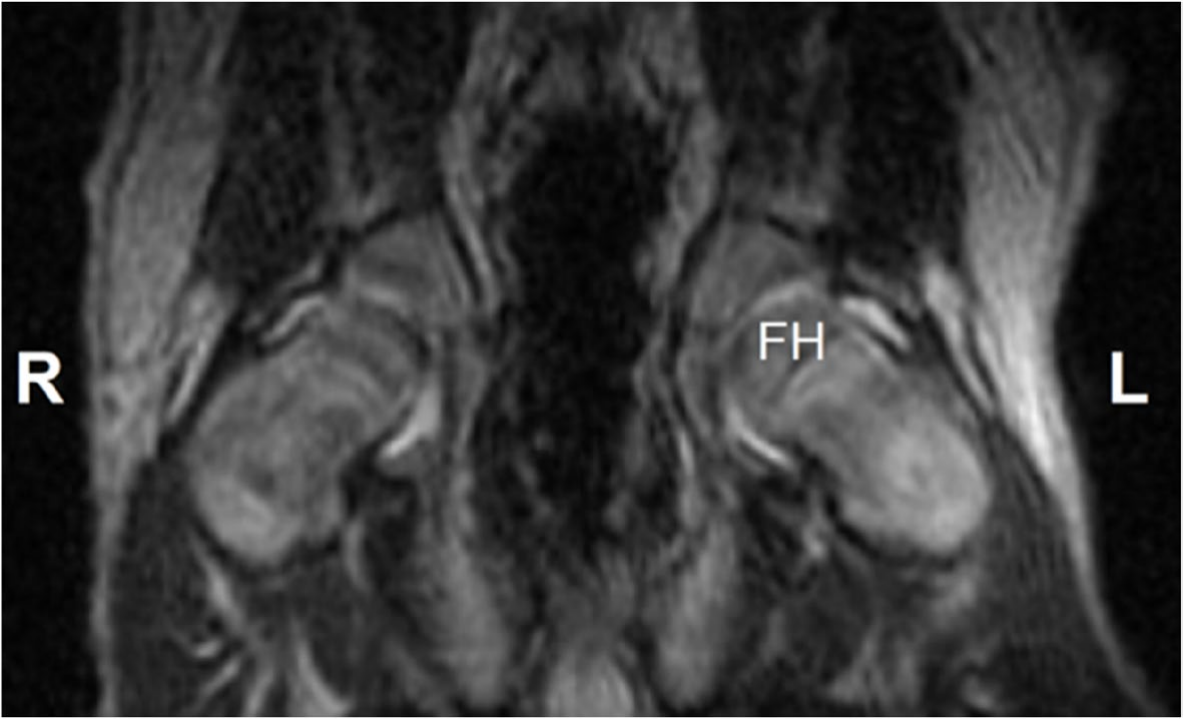
Dorsal T2-weighted magnetic resonance image of a 2-month-old Estrela Mountain Dog dog that developed severe hip dysplasia as an adult. The image shows the cranial and caudal recesses of the synovial membrane, in the right (R) and left (L) hip joints, with high-intensity signal due to their synovial fluid content. FH: left femoral head

Lust et al. [109] have determined that increased articular volume, in young dogs genetically predisposed to HD, is correlated with greater joint laxity and subluxation. Ginja et al. [67] have drawn similar conclusions, suggesting an association between an early increase of the hip synovial fluid volume, in 7- to 9-week-old puppies assessed by magnetic resonance imaging, with later development of HD (Fig. 4). Moreover, the quantification of early changes in the synovial fluid markers may be also of interest in terms of disease screening or prevention [114].

Concerning the treatment options, both conservative and non-conservative management are available and shift throughout the skeletal maturity of the dog. Conservative treatment is often considered the first-line therapy at the onset of HD clinical signs, ensuring the relief of discomfort and pain, the preservation of limb function and range of motion, and the improvement of life quality [95, 115]. In immature dogs, conservative treatment entails limiting intense and painful exercise, weight control, physical therapy, and administration of analgesics [115] or nutraceuticals [48]. Slight, low impact and high-resistance exercise, based on off-leash walking [116] or swimming are recommended [117], as these exercises improve muscle mass strength and joint range of motion [118]. In mature canines, it is centred on treating OA-related pain, by using non-steroidal anti-inflammatory drugs [18, 119]. Hence, recent advances have suggested a novel therapy with anti-nerve growth factor monoclonal antibodies as a replacement for traditional analgesics [120, 121]. Furthermore, intra-articular administration of hyaluronan [122, 123], corticosteroids [122], platelet-rich plasma [124], or ozone gas [123] are also considered an alternative when controlling joint pain and inflammation. In addition, the wide range of early mechanical conservative options available in humans, lack in dogs. The non-easily application of coaptation devices on ambulatory animals and the late HD diagnosis, in dogs, may explain the absence of closed hip reduction methods in the canine specimen [18]. Riser and Shirer [125] attempted to promote hip congruence by maintaining young dogs in small cage confinement in an abducted-flexion position. However, the absence of social development led to the abandonment of this approach [125]. Undoubtedly, the success achieved in the last few years on account of human closed-reduction methods should be set as an example to be followed by veterinarians in the future.

Regarding non-conservative management, surgical alternatives for young animals are designed as a preventive measure, ensuring improved joint alignment and joint laxity, and limiting the progression of OA. These surgical alternatives include juvenile pubic symphysiodesis [85, 126] and pelvic osteotomies [85]. For dogs displaying symptomatic disability and pain with severe degenerative joint disease, joint capsular denervation [127, 128], femoral head and neck ostectomy, and total hip replacement are the existing salvage surgical procedures [85]. Nevertheless, preventive surgical options, in the most severe cases, may not be as effective long-term [129], since total hip replacement is the only procedure capable of restoring the lost hindlimb function in certain stages of HD [130].

Overall, the effectiveness of canine screening, breeding programmes, and treatment outcomes are remarkedly lower when compared to the success accomplished in human medicine in the last decade regarding the progress of HD approaches. Otherwise, the prevalence of canine HD would have decreased considerably. Yet, the issue remains and questions regarding the effectiveness, reach, and homogeneity of the screening programmes, and the weight of the genetic and environmental components are raised. The fact that neglected HD cases are common and only a minority of dogs have access to the mentioned surgical procedures should raise awareness of the urgency of addressing this medical condition among tutors, breeders, and veterinarians.

### Animal models for hip mechanical osteoarthritis

Animal modelling usage dates to the sixth century before Christ and their use in the pursuit of biomedical research has persisted ever since [131]. When studying hip MOA, two types of animal models are described in the literature, experimental induced models [132] and natural or spontaneously occurring models [11, 132]. The last models are more likely to mimic human OA due to the slower onset and progression of the disease. However, the accessibility of such models represents a current limitation [132].

### Experimental induced animal models

A panoply of small and large animals is available for induced models of MOA [20]. Small animals, as rodents and rabbits, have relatively low maintenance due to their small size, practicality to house, and cost when compared to large animals [20, 133, 134]. Both have contributed to improving our perception of the disease physiopathology, despite large animals being recognised for fostering more relevant data [134, 135]. Large animal models, such as dogs, sheep, goats, or horses, have more similarities in joint biomechanics and cartilage thickness and structure [134–136]; allow the collection of synovial fluid and therapeutic intraarticular administrations; and are amenable to diagnostic imaging [135, 136], and post-surgical management [20, 135, 136]. Still, due to ethical concerns, small animal models are most commonly employed in initial trials and screening studies [134]. Likewise, large animals take longer to skeletally mature [136].

The rabbit has been very popular for years as an animal model of Human hip MOA [137–143], regardless of the differences in gait, biomechanics, and structural variations in the cartilage thickness and chondrocyte density [134]. According to Arzi et al. [144], rabbits demonstrate a relationship between OA and obesity as observed in humans, along with an analogous disease progression pattern. This species, as a spontaneous OA model, can allow a foreseeable translation of findings in bioengineering studies concerning the naturally arising disease in humans [144].

The first rabbit model of hip MOA was described in 1956 [143]. To date, several rabbit models of hip MOA induction are centred on 1 to 8-week-long hindlimb immobilization with the knee in extension (Table 1) [137–143]. The methodology used is efficient in inducing luxation/ subluxation [137–142] and the development of degenerative joint disease [142, 145], yet causes long-lasting malfunctions in animals [140]. The animals are unable to use the pelvic limb due to the reduced flexion of the stifle and long-standing luxation will lead to permanent tissue metaplasia and, therefore, to the loss of the cartilage remodel potential. Moreover, the limb becomes permanently disabled, which makes these types of models unfit to test therapeutic solutions in vivo [140].

**Table 1:** Rabbit models of hip mechanical osteoarthritis using the knee in extension

Type of Immobilization	Sample Size (limbs)	Rabbits’ Age (days)	Immobilization’s Duration (weeks)	Sequela	References
Knee extension using a compression arthrodesis device	-	-	-	Increased cartilage thickness Sclerotic thickened trabecula Bone cysts Degenerated disintegrating articular cartilage	[143]
Knee extension, right limb with a splint and left limb with a padded plaster-of-Paris splint	-	42 – 56	6	Excessive femoral retroversion Round ligament hypertrophy Posterior Capsule Inversion Oedema Capsular fibrosis	[141]
Plastic tube with the knee extension (one/both limbs)	*n* = 87	7 – 56	1—8	Luxation Subluxation Coxa Vara	[139]
Unilateral knee extension using a Kirschner wire	*n* = 60	7 – 21	-	Luxation Subluxation	
Right knee extension using a cast	*n* = 7	~ 19	1.5	Luxation Flattening of the articular cartilage Permanent tissue metaplasia	[140]
	*n* = 6	~ 33	1.5		
Left knee extension using a cast plaster	*n* = 9	28	2	Increased cartilage thickness Capsular fibrosis Chondrocyte necrosis	[142]
	*n* = 9		4		
	*n* = 9		6		
Unilateral knee extension using a cast plaster	*n* = 6	28	2	Chondrocyte apoptosis	[137]
	*n* = 5		4	Chondrocyte apoptosis Subluxation	
	*n* = 5		6	Luxation	

Long-term immobilization of the hindlimb with knee extension in young rabbits easily results in the permanent loss of limb functionality, and reduced range of motion [146], causing friction-induced skin injuries, patellar luxation, joint stiffness and, distally, oedema and ischemia. Regarding research in HD therapeutic solutions, animal models of hip MOA are scarce or non-existent. Hence, the development of a functional animal model of hip MOA would fulfil its current need in the veterinary and human HD research fields. Nevertheless, animal experimentation has raised some ethical concerns and should follow the “4Rs” principle (Reduction, Refinement, Replacement, and Responsibility), growing awareness of the need to explore robust alternatives and guarantee responsible research practices [147].

### The dog as a natural animal model in a one medicine concept

**Fig. 5:**
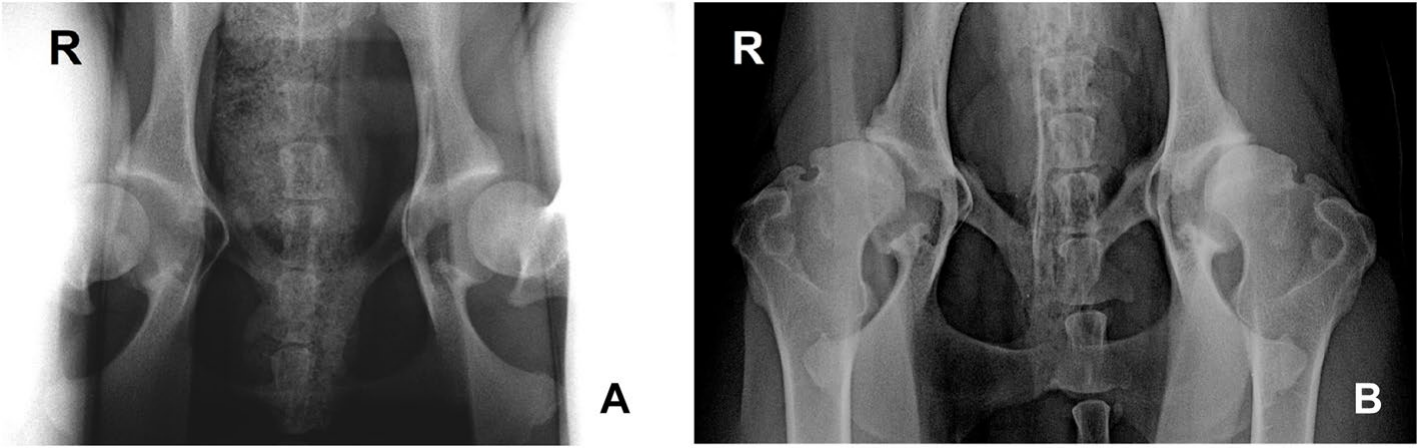
**A** Stress hip radiograph of a 10-month-old dog showing a bilateral increase in the hip joint laxity. **B** Ventrodorsal hip extended view of the same dog 10-months later, at the age of 20-months-old, displaying radiographic signs of bilateral severe degenerative joint disease in the acetabulum and femoral head due to bone remodelling and osteophyte development

One Medicine is an emerging concept joining veterinary and human medical professionals for an improved and more comprehensive understanding of the naturally occurring MOA, which presents strong homologous aspects in both congenital and canine HD [18]. Canine HD remains with a high prevalence in some breed populations, due to different reasons, breeding programs based on animal selection are not always implemented or fail to achieve the desired success [26]. The dog is considered the nearest to humans in terms of OA development, anatomic resemblance, disease heterogeneity [135], and responses to conventional conservative and surgical treatments [20, 135]. In naturally occurring OA, humans and dogs also share environmental and genetic traits, namely gender [118, 148, 149], breed [118, 150]/ethnicity [151], obesity [118, 152], diet [153, 154], and early joint laxity followed by hip instability and gradual OA development [155–157] (Fig. 5).

The dog is a potentially available natural animal model presenting some unique advantages over induced animal models, as it mimics the gradual development of human hip MOA associated with joint laxity and instability [17]. Both species have equivalent life stages, being about 5 times faster in dogs, allowing a longitudinal evaluation of the spontaneous disease in a shortened period and, also, post-mortem retrieval studies [11]. An additional advantage of OA spontaneous models results from an overall reduction in the number of animals needed for experimental purposes which are aligned with the 3Rs principle [158]. On the other hand, we should not be oblivious to the fact that the dog itself can benefit from many human cutting-edge therapies [11] and from a natural model of OA perspective, can become the main beneficiary. Nevertheless, natural disease models differ from similar experimental animal models as longitudinal follow-up should be non-invasive, non-harming and research groups need to be larger, to compensate for the diversity of environmental factors and loss of follow-ups in some animals, and to achieve a suitably powered study design [11]. Additional concerns from animal models of OA come from the difficulty of quantifying the degree of OA-related pain compared to humans and dogs would greatly benefit from complementary means of objective analysis such as pressure platforms [159, 160] or non-invasive imaging methodologies [161, 162]. A patient-centred approach should be implemented with inclusion of outcome surveys to obtain follow-up information about pain-related behavioural expression in dogs [163, 164].

Dogs are the animal species described with the highest natural predisposition for the development of HD [18, 83, 84] and, as a result, hold the greatest potential for the identification of equivalent disease loci, homologous genes, and biochemical mechanisms in human developmental HD [17]. Therefore, the creation of a multidisciplinary consortium between veterinary, human physicians and biologists would allow an exchange of expertise and knowledge in the area of OA [17], similar to what has already been achieved in other fields [165, 166]. Furthermore, veterinary biobanks accept biological samples from osteoarthritic dogs [11], which later can be used for biomarkers discovery, supporting the use of the dog as the optimal translational model of human HD [167].

## Conclusion

Hip MOA is a daily clinical reality in different animal species, being usually reported and more challenging in humans and dogs, affecting greatly their health, well-being, and quality of life. In both species, the development of hip OA has many mechanical resemblances. Nevertheless, some important differences should be taken into consideration when extrapolating data from both species, namely the type of locomotion (biped/ quadruped) and the disease onset (congenital/non-congenital). In human hip MOA, preventive methodologies associated with an early diagnosis have been applied with great success, limiting the disease progression. Canine HD screening, breeding programs, and treatment outcomes have considerably lower success when compared to humans. Following the One Medicine concept, the accomplished success in human preventive treatments for HD should be seen as an encouragement for veterinarians and researchers to pursue more effective and innovative procedures accessible and affordable to all dogs. Research in HD functional animal models may lead to additional acknowledgement regarding therapeutic approaches. Moreover, One Medicine is an emerging concept joining the veterinary and human medical expertise for a better acknowledgement of shared diseases and, the dog as a spontaneous model of MOA, may be used in translational research.

## Data Availability

Not applicable.

## Abbreviations

MOA: Mechanical osteoarthritis
HD: Hip dysplasia
OA: Osteoarthritis
OARSI: Osteoarthritis Research Society International
ERDF: European Regional Development Fund
OPCI: Operational Programme for Competitiveness and Internationalization
FCT: Portuguese Foundation for Science and Technology
